# Nanodiagnostics to Face SARS-CoV-2 and Future Pandemics: From an Idea to the Market and Beyond

**DOI:** 10.1021/acsnano.1c06839

**Published:** 2021-10-27

**Authors:** Giulio Rosati, Andrea Idili, Claudio Parolo, Celia Fuentes-Chust, Enric Calucho, Liming Hu, Cecilia de Carvalho Castro e Silva, Lourdes Rivas, Emily P. Nguyen, José F. Bergua, Ruslan Alvárez-Diduk, José Muñoz, Christophe Junot, Oriol Penon, Dominique Monferrer, Emmanuel Delamarche, Arben Merkoçi

**Affiliations:** † Institut Català de Nanociència i Nanotecnologia, Edifici ICN2 Campus UAB, 08193 Bellaterra, Barcelona, Spain; ‡ MackGraphe-Mackenzie Institute for Research in Graphene and Nanotechnologies, Mackenzie Presbyterian University, Consolação street 930, 01302-907 São Paulo, Brazil; § ISGlobal-Barcelona Institute for Global Health, Carrer del Rosselló, 132, 08036 Barcelona, Spain; ∥ Université Paris-Saclay, CEA, INRAE Departement Médicaments et Technologies pour la Santé SPI, 91191 Gif-sur-Yvette cedex, France; ⊥ Asphalion, Carrer de Tarragona 151-157, 08014 Barcelona, Spain; # IBM Research−Zurich, 8803 Rüschlikon, Switzerland

**Keywords:** COVID-19, SARS-CoV-2, nanodiagnostics, biosensors, bottlenecks, outbreaks, testing methods, phases of test development

## Abstract

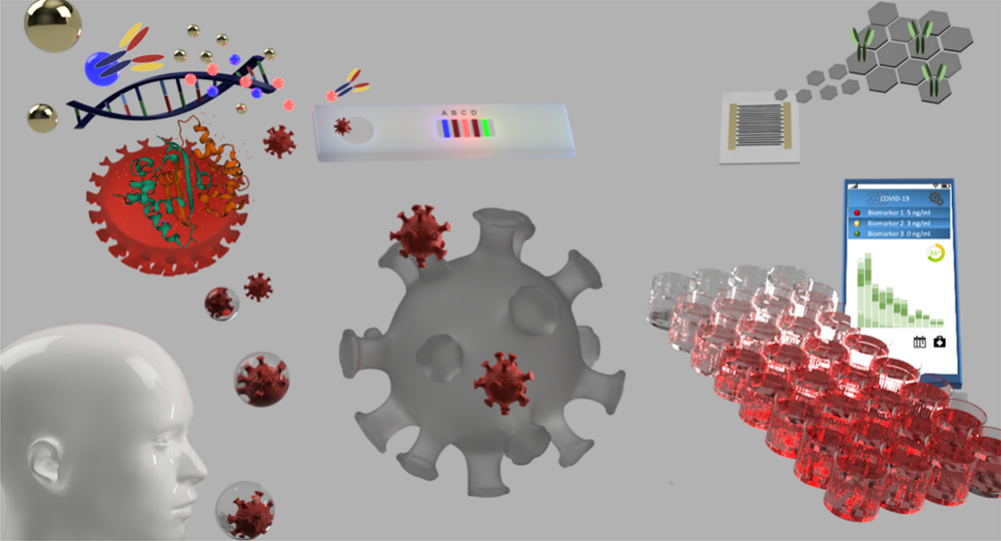

The COVID-19 pandemic made clear how our society requires quickly available tools to address emerging healthcare issues. Diagnostic assays and devices are used every day to screen for COVID-19 positive patients, with the aim to decide the appropriate treatment and containment measures. In this context, we would have expected to see the use of the most recent diagnostic technologies worldwide, including the advanced ones such as nano-biosensors capable to provide faster, more sensitive, cheaper, and high-throughput results than the standard polymerase chain reaction and lateral flow assays. Here we discuss why that has not been the case and why all the exciting diagnostic strategies published on a daily basis in peer-reviewed journals are not yet successful in reaching the market and being implemented in the clinical practice.

During the last decades and even more specifically during the last few months, we all became familiar with the term “diagnostics”, which includes devices and methods used for identifying a particular disease. Diagnostic devices and assays are now routinely used by medical doctors as a valuable aid for the diagnosis of the patient. In fact, once we analyze what it would take to perform a diagnosis without a diagnostic device, we find many variables to take into account, making an accurate diagnosis far from being simple. For example, with the coronavirus 2019 (COVID-19) pandemic, medical doctors have to diagnose a patient that shows influenza-like symptoms. For this, they would have to consider factors such as the prevalence of a specific disease during that time of the year (*i.e.*, flu season or not), the severity of the symptoms, the overall status of that specific patient, chronic or seasonal conditions (i.e., allergies), general patient behavior (*i.e.*, travels, vaccinations), *etc*. Performing this type of assessment for each patient takes time (which may be limited during a pandemic or in busy hospitals), and, above all, it is heavily subjective. On one hand, the doctor needs to rely on the answers of the patient, which may differ from individual to individual experiencing the same symptoms. On the other hand, even the best doctors can diverge in the diagnosis of the same individual. In this context, diagnostic tests reveal themselves as great tools to help medical doctors to precisely diagnose patients at the first visit, without additional examinations.

Imagine what can be done with a portable, low-cost sensing device that could be able to rapidly detect in a single step any arbitrary infectious agent in a biological fluid. Such a technology would revolutionize our current approach for handling infectious diseases, because it could enable nearly real-time detection of a pathogen at the population level. This would have multiple positive repercussions: (1) it would allow decreasing the number of undetected cases; (2) it would improve our knowledge of the epidemiology of the disease; (3) we could make timely decisions on the treatment of infected patients, maximizing their chances of recovery; (4) we could more effectively control the propagation of the disease. As a result, this could dramatically impact the public health system and economy of whole countries via more granular and timely measures such as lockdowns and border closings. In this manuscript, taking COVID-19 diagnostic as model, we analyze every step (from the definition of the idea to the market placement) that can stop or slow down the development of a diagnostic device.

## The SARS-CoV-2 Pandemic and Other Recent Outbreaks

SARS-CoV-2 is a human-pathogenic strain of coronavirus that was discovered in Wuhan (Hubei Province, China) at the end of 2019.^1^ SARS-CoV-2 stands for severe acute respiratory syndrome-related coronavirus 2 and is responsible for the COVID-19 disease, which causes from mild to severe respiratory symptoms in humans.^2^ By October 27th of 2021, SARS-CoV-2 has officially infected more than 243 million people and killed 4 953 246 people worldwide.^3,4^ The high infectivity of the virus is due to its ability to be transmitted through the direct contact with droplets produced by sneezes or coughs of infected people (thus involving airborne transmission, more probable in closed spaces) and indirectly through contact with contaminated surfaces (World Health Organization - WHO indications). The reproduction number (R0) gives a measure of the infectivity of a virus, representing the expected number of new cases in a community directly generated by an infected person. Initially, the R0 of SARS-CoV-2 was estimated between 1.4 and 3.9.^5^ However, with the emergence of the Delta variant this value increased almost to 7.^6,7^ While the first steps for containing an outbreak are to identify transmission pathways and estimate the R0, the management of a pandemic also requires knowing the infectious agent structure and genome in order to develop effective diagnostic tools in the shortest possible time.

For diagnostic purposes, in support of the clinical examinations, the first step is the identification of a specific biomarker, the concentration of which should vary sensitively and concurrently in the presence of the infection. In the case of SARS-CoV-2, as for other viruses, we can identify two major classes of viral biomarkers: nucleic acids and proteins. Regarding the former, as for all coronaviruses, SARS-CoV-2 has a positive-sense single-stranded RNA (+ssRNA) that allows for a direct translation of the viral genetic material into viral proteins within the cytoplasm of the infected cells.^5,8^ Regarding the latter, SARS-CoV-2 displays four structural proteins, named as the spike protein (S protein), the envelope protein (E protein), the membrane protein (M protein), and the nucleoprotein (N protein).^9^ The S, E, and M proteins are actually glycoproteins present on the outer membrane of the virus, whereas the N protein holds the genome within the viral particle. The S protein is composed of two subunits named S1 and S2. During the infection, the subunit S1 enables the attachment of the virus to the host cell, whereas the subunit S2 triggers the internalization of the virus within the cell.^10,11^ Then, the E protein, which is the smallest structural protein of SARS-CoV-2, helps the dissemination and replication within the host cell.^12^ Next, the M protein is the main structural protein of the viral membrane and determines the shape and size of the virion, stabilizing the nucleocapsid and promoting the overall assembly.^5^ Eventually, the N protein is involved in the viral replication and triggers the cellular immune response of the host against the virus.^13^ Because of the ability of the virus to spread and circulate throughout the body, these biomarkers have been found in several biological fluids, including saliva, blood, stool, feces, and semen of infected people.^14,15^

**Figure 1:**
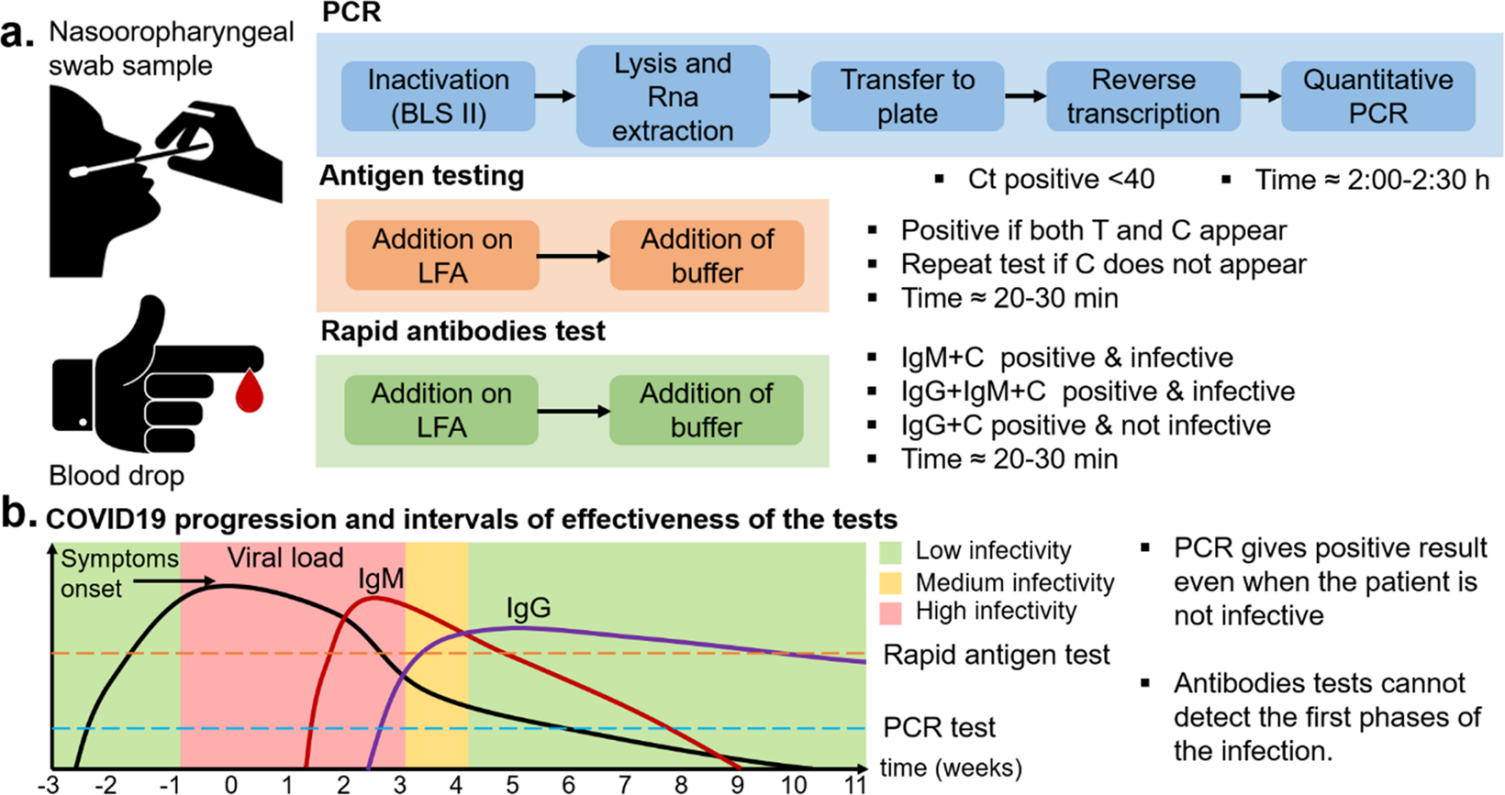
Visual description of the duration of the phases required to develop a rapid test for previous outbreaks (the dates refer to each phase thanks to a color code) (a) and of the current SARS-CoV-2 detection technologies (b). PCR, antigen, and serological tests with the respective criteria for results interpretation (T = test line, C = control line). The bottom plot gives an outlook of the infection progression and the phases in which each test result is more effective.

Looking back to other viruses’ outbreaks, we can observe many efforts done to improve the time required for the development of diagnostic approaches. Indeed, if we compare the 2003 SARS-CoV, 2009 A/H1N1, 2014 Ebola, and 2019 SARS-CoV-2 outbreaks, we can observe a reduction in the reaction time toward the monitoring and estimation of infected cases during the epidemics (Figure 1a). This quicker response is mainly due to the technological advances achieved over the last 20 years on DNA sequencing, which have allowed us to save time in the identification of clinically relevant biomarkers as well as to improve the performance of molecular kits based on the nucleic acid detection (**e.g.**, polymerase chain reaction (PCR) and isothermal amplification techniques). Although there are several factors involved in the spreading of an epidemic, such as the mortality of the pathogen, the geographical impact, or the economic resources of the affected countries, the use of these molecular diagnostic tools allowed the World Health Organization (WHO) and the other national agencies to agree in the implementation of the same guidelines to control the pandemics. More specifically, these actions are based on the achievement of frequent testing: pathogen isolation/identification, pathogen sequencing, the design and release of a PCR protocol (namely, molecular kits), and the development of rapid tests.

The ability to sequence the viral genome influenced deeply also the discovery of protein biomarkers. Indeed, thanks to current molecular biology techniques, we can now mass-produce virtually any protein as soon as its sequence is identified. This allows the selection of suitable bioreceptors (*e.g*., antibodies and aptamers) for the development of several laboratory-based assays (*e.g*. enzyme-linked immunosorbent assay (ELISA) and western blots) and point-of-care (POC) systems (*e.g*., lateral flow assay (LFA), electrochemical sensors, and microfluidic devices).^16−18^ For example, only 43 d after the publication of the SARS-CoV-2 genome, two different LFAs for the detection of SARS-CoV-2-specific antibodies were being distributed in China under an Emergency Use Approval (EUA) authorization.^19^ Thanks to their low cost, easy operation, and fast response compared with PCR, the use of LFAs has allowed the decentralized high-frequency testing of millions of individuals leading to an effective containment of the virus spread.^20^

Despite the COVID-19 outbreak having been faced with the quickest global response, regarding diagnostic devices, much still needs to be done if we want to prevent the damage caused by future pandemics. Specifically, current techniques for the detection of a nucleic acid are still too slow (∼1 h), cumbersome (multistep), and expensive (requiring costly equipment) to be effectively deployed at the point of care.^21^ Instead, antigenic POC tests based on the detection of protein biomarkers (*i.e*., antigens and antibodies) are now widely used and deployed in order to highlight a positive case and thus limit virus transmission.^22,23^ Fortunately, during the last decades, researchers have been publishing exciting methods and technologies that address those issues, and their further development into commercial products could revolutionize the way we diagnose diseases.^24,25^

## Current Testing Methods

The diagnosis of COVID-19 currently relies on the detection of two viral biomarkers (*i.e.*, viral RNA and antigens) and one host biomarker (*i.e.*, SARS-CoV-2-specific antibodies). Each target can provide different information about the clinical status of the patient. Generally, the SARS-CoV-2 virus clinically relevant concentrations are between 10^2^ and 10^11^ viral particles per milliliter in saliva.^26,27^ The virus RNA is indirectly detected by means of molecular techniques, with the quantitative reverse transcription polymerase chain reaction (qRT-PCR) being the most used. As depicted in Figure 1b, with this technique, RNA is converted into a complementary DNA chain by an RNA-dependent DNA polymerase (reverse transcriptase) and is subsequently amplified by a DNA polymerase following thermal cycles. Primers (*i.e.*, nucleic acids used for the initiation of the DNA synthesis), probes, and the transcribed DNA are required to perform qRT-PCR; therefore, it is necessary to know the genome sequence beforehand. The SARS-CoV-2 genome sequence was reported 10 d after announcement of the outbreak in China,^28,29^ and the first PCR tests were produced and distributed after less than two weeks.^30^ The main disadvantages of qRT-PCR tests are the turnaround time required to obtain a result (not shorter than 1 h, with commercial assays such as GenXpert or Filmarray)^31^ and the need for skilled personnel and expensive laboratory equipment.^32^

Rapid antigen tests, such as LFA, represent one of the most recognized diagnostic tools used during this pandemic to perform a fast screening. Basically, they are based on the same sampling method used for PCR, but the swab is introduced in the LFA with the use of a working solution, which let the sample flow through a paper membrane. When the solution comes in contact with the sample pad, the viral particles are recognized by nano- or microparticles (*e.g.*, gold nanoparticles (AuNPs), latex beads) functionalized with specific antibodies that recognize the proteins of the virus membrane. The AuNPs-virus complexes are then trapped by secondary SARS-CoV-2 binding antibodies immobilized on the test line, and their accumulation generates a visible color (positive test). The unbound AuNPs are recognized by the control line, which permits the test administrator to distinguish a negative test (only control line present) from an unsuccessful test where, for example, the solution did not flow properly (no line present). Although a rapid antigen test can provide results in a few minutes, they are less sensitive than PCR, often leading to false-negative results. Information about the analytical performance of such devices based on LFA technology can be found in the FIND database, as highlighted at the end of this section.

Rapid tests for measuring the immune response of patients are also mostly based on LFAs. These tests, often referred to as serological tests, permit a determination of the presence and type (mostly IgA, IgG, or IgM) of SARS-CoV-2-specific antibodies.^32^ The sensing mechanism is similar to those of antigen-based LFA; however, the targets of the test are not the viral particles but the IgG and IgM antibodies, and the sample used can either be a drop of blood (for IgG and IgM, with two test lines) or saliva (for IgA and IgG). Although a serological test cannot be used for an early diagnostic, because the immune system requires several days to develop an immune response strong enough to be accurately measured, they can provide complementary diagnostic information on patients having symptoms and negative rRT-PCR results,^33^ and they are particularly useful to monitor the patient’s immunity acquired either via an infection or vaccination.

Because each biomarker can describe a different phase of the infection process, the estimation and relative concentrations of biomarkers strongly depend on the time chosen for the test, as highlighted in Figure 1b. More specifically, PCR testing is effective in detecting the virus 10 d after the infection occurred and until the sixth week after it. Antigen tests are usually effective in detecting it between the second and the fifth week, while serological tests can detect the infection only three to four weeks after the infection started.^34^ Generally, there are two main parameters used to define the performance of a diagnostic test: sensitivity and specificity. They refer to the proportion of true positives or true negatives when being compared to a standard technique (here PCR), respectively.^35,36^ Thus, a final interpretation of a diagnostic test is not just given by its operating features but also for the prevalence or pretest probability of disease.^31^

Recently, the Foundation for Innovative New Diagnostics (FIND) set up a database of the commercially available COVID19 diagnostic devices, which is updated on a regular basis. Of the current 544 diagnostic devices available (data updated May 2nd, 2021), 158 are based on RNA detection and 276 on antigen detection, and 101 are serological. FIND also conducts the independent evaluation of these commercially available technologies, comparing them analytically by their effective specificity, sensitivity, and response time and giving many additional information such as the type of sample used, the connectivity, *etc*. Both the database and the results comparison are publicly available in the FIND Web site.^37^

## The Nanodiagnostic Devices Scenario

The ideal characteristics of a POC test for antigen detection are described and summarized by the REASSURED criteria coined by Land and Peeling *et al*.^38^ Specifically, it states that a sensor should provide (i) real-time connectivity, (ii) ease of specimen collection, with all the required protocols or sample treatment steps integrated in the device. The device should also be (iii) affordable, (iv) sensitive, (v) user-friendly, (vi) rapid and robust, (vii) equipment-free, and (viii) deliverable to end-users. Important efforts are currently directed to the improvement of these diagnostic devices. Furthermore, research groups from all over the world are proposing innovative solutions based on nanomaterials to radically decrease the minimum detectable antigen concentrations and thus the time gap between infection and diagnosis.

The peculiar characteristics of nanomaterials (materials with features of 100 nm or smaller) have the potential to dramatically improve the performance of the diagnostic device toward achieving the REASSURED characteristics.^39^ In fact, nanomaterials have properties that differ from those of the same materials at the macroscopic scale, showing phenomena such as quantum confinement, electromagnetic field enhancement, and signal amplification. Consequently, these can be harnessed to include signaling and recognition phenomena in diagnostic devices, such as narrow emission band fluorescence, surface plasmon resonance, and conductivity.^40,41^ The nanometer size enables the performance of analytical measurements with high functionality and sensitivity.^42^ For example, zero-dimensional (0D) nanomaterials such as nanoparticles are the basis of LFAs, transducing the color change of the test and control lines in the presence of the analyte.^43^ Furthermore, nanoparticles allow the increase of electrochemical sensors surface area and the control of their functionalization with bioreceptors such as DNA, aptamers, and antibodies.^44,45^ Two-dimensional (2D) nanomaterials such as graphene permit the fabrication of ultrasensitive semiconductors functionalized with bioreceptors for the specific detection of small molecules, proteins, and DNA.^45−48^

**Figure 2:**
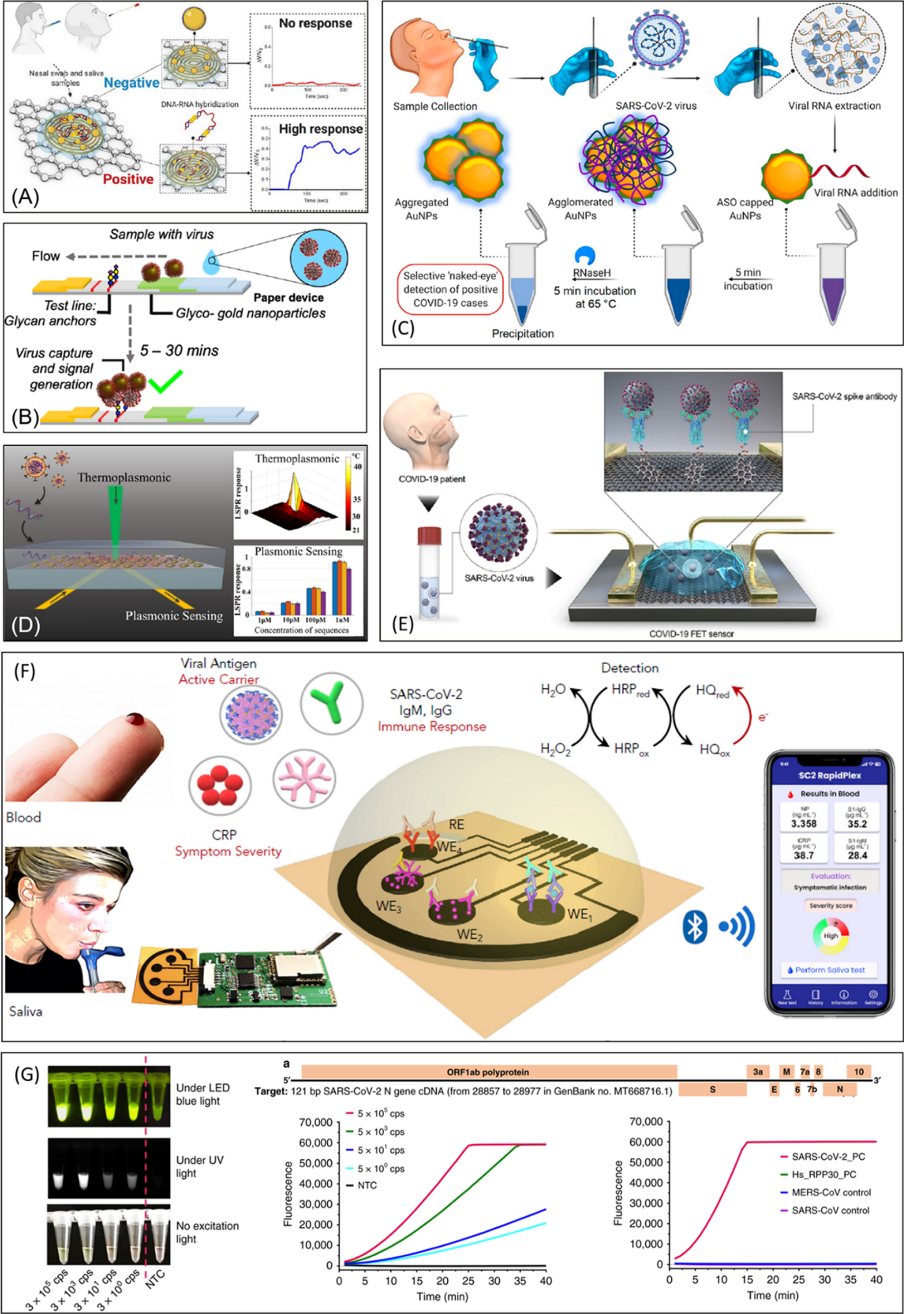
Examples of nano-biosensors for the detection of the SARS-CoV-2 virus and published in 2020. (A) Electrochemical paper-based interdigitated device for N gene detection using graphene and AuNPs.^52^ Adapted with permission from ref (52). Copyright © 2020 American Chemical Society. (B) Spike protein lateral flow device using AuNPs and glycan anchors.^53^ Adapted with permission from ref (53). Copyright © 2020 American Chemical Society. (C) Colorimetric (plasmonic) viral RNA detection method with the use of DNA-functionalized AuNPs.^56^ Adapted with permission from ref (56). Copyright © 2020 American Chemical Society. (D) Thermoplasmonic device for the detection of membrane, spike, and nucleocapsid protein genes with DNA immobilized on 2D nanoislands.^57^ Adapted with permission from ref (57). Copyright © 2020 American Chemical Society. (E) Graphene-based FET for real-time immune-based spike protein detection.^58^ Adapted with permission from ref (58). Copyright © 2020 American Chemical Society. (F) Multiplexed electrochemical detection on laser-engraved graphene electrodes.^60^ Adapted with permission from ref (60). Copyright © 2020 Elsevier. (G) CRISPR-based ultrasensitive fluorescent detection of two sites of the viral RNA genome.^64^ Adapted with permission under a Creative Commons Attribution 4.0 International License from ref (64). Copyright © 2020 Springer Nature.

Besides nanomaterials, another key nanoscience area with a huge potential to improve the performance of diagnostic test is nano-biotechnology. For example, the selection of more effective bioreceptors (*e.g*., nanoswitching DNA fragments, aptamers, nanobodies) can contribute enormously to the enhancement of sensing performances;^49−51^ this can be seen very well in the recent work of Idili *et al*.,^51^ in which the authors showed the development of an electrochemical aptamer-based (EAB) sensor able to achieve the rapid, reagentless, and quantitative measurement of the SARS-CoV-2 spike protein. These technologies are well-understood from a theoretical and proof-of-concept level and need now to be adapted for a more practical sampling and ensure a specific detection, quantitative and portable measurement and analysis, rapid response time, and high reliability. In Table 1 we report some of the latest and most promising nanotechnological devices for SARS-CoV-2 detection, illustrating in Figure 2 some of the respective detection mechanisms.

**Table 1:** Comparison of Some of the Most Promising Results Available Currently in the Scientific Literature for Nanomaterial-Based Diagnostic Devices

analyte	receptor	nanomaterial	transduction	LoD	resp time	ref
Nucleocapsid phospho-protein (N gene)	DNA	Graphene paper-based device decorated with AuNPs	Electrochemical interdigitated electrodes	6.9 cP/μL	5 min	Figure 4A^52^
S1 Spike glycoprotein	N-acetyl neuraminic acid	Glyco-gold nanoparticles	Colorimetric with lateral flow assay	5 μg/mL	30 min	Figure 4B^53^
IgG, IgM and antigen	Antibodies	AuNPs	Fluorescence		15 min	(54)
S1 Spike glycoprotein	Membrane engineered mammalian cells	Membrane modified cells	Bioelectric recognition assay	1 fg/mL	3 min	(55)
Nucleocapsid phospho-protein (N gene)	Antisense oligo-nucleotides	AuNPs	Optical colorimetric (plasmonic)	0.18 ng/μL	10 min	Figure 4C^56^
Membrane, Nucleocapsid and spike protein genes	DNA	2D gold nanoislands	Plasmonic photothermal effect	0.22 pM		Figure 4D^57^
S1 Spike glycoprotein	Antibody	Graphene	FET-based detection	1 fg/mL (S1) 2.42e2 cP/ml (virus)	Real time	Figure 4E^58^
S1 Spike glycoprotein and Nucleocapsid protein	Antibody	Semiconductor single walled carbon nanotubes (sc-SWCNTs)	FET-based detection	0.55 fg/mL(S1) and 0.016 fg/mL(N)	2 min	(59)
Nucleocapsid protein, IgG, IgM, C-reactive protein	Antibodies	Laser engraved graphene	Electrochemical		1 min	Figure 4F^60^
Viral RNA	DNA	Graphene and AuNPs	Electrochemical	200 cP/ml	3 h	(61)
ORF1ab, N gene	DNA/Antibodies	AuNPs	LAMP lateral flow assay	12 cP/reaction	1 h	(62)
Volatile organic compounds (VOC)	Organicligands	AuNPs	Chemiresistors–breath sensor	Accur. 95%	19 s	(63)
Two viral RNA target sites	Cas12a enzyme	none	Fluorescence	5 cP/reaction	20–40 min	Figure 4G^64^

## The Phases of Diagnostic Devices Development

The complete development of a diagnostic device, from its conception to the market, takes, on average, from 3 to 5 years.^65^ Despite a faster time compared to the commercialization of a drug (normally taking from 12 to 15 years) due to the need of less preclinical and clinical data for the regulatory aspects, safety and performance validation remain key milestones in every diagnostic device development. The recently coined Technology Readiness Level (TRL) defines a score system identifying the key milestones during the development of a diagnostic device. TRL incrementally ranges from 1 (observation of the fundamental principles of the device technology) to 9 (real system tested in operative environment).

Typically, the research and the development of a diagnostic device takes slightly different ways with respect to which type of entity is developing it. In fact, even with some exceptions, business-related entities (*e.g.*, spin-off, small and medium-sized enterprises (SMEs), and/or big companies in the sector) are more focused on the innovation related to the application. In this case, even a slight improvement with respect to the previously available technologies is considered relevant having a potentially strong economic impact. Conversely, academic research entities but also parts of the research and development (R&D) divisions of big companies in the sector are more interested in the discovery of novel detection mechanisms and mainly pursue the reduction of the detection limits of the currently available diagnostic devices. Although these look like opposite directions, they are actually two faces of the same coin, and they both represent innovation.

**Figure 3:**
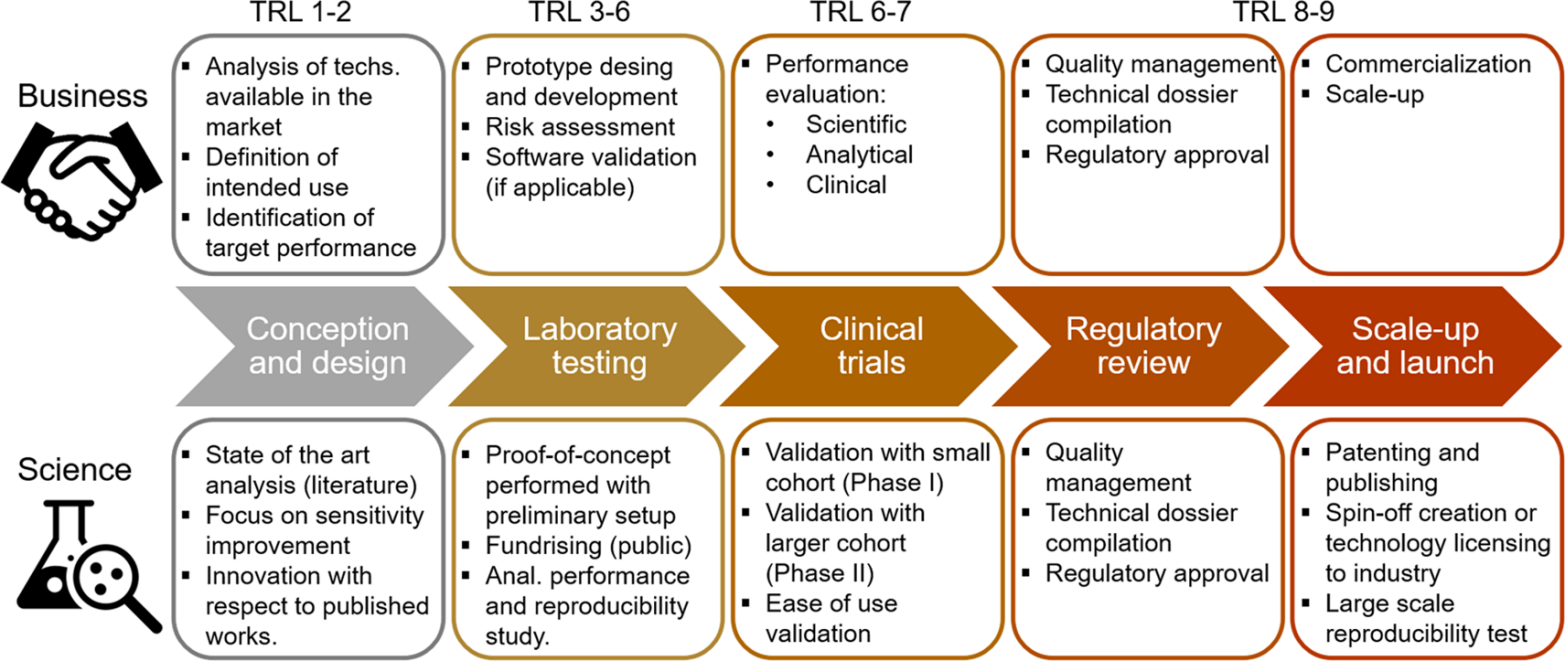
Current development process of diagnostic devices with the phases from conception to the market launch and corresponding TRLs.

The phases of the development of a diagnostic device, both from the business and research points of view, are summarized in Figure 3, accompanied by their respective TRLs and discussed in the following paragraphs.

### Conception and Design

The first action to be taken to develop a diagnostic device is a precise definition of the problem that will be solved through this sensing technology. The problem must have specific and quantifiable characteristics, such as impacting the lives of a significant number of people, represent a new or severe disease, which early diagnosis is essential to improve the recovery rate of patients, be a disease with a high transmission rate, *etc.*(24) The design of a diagnostic device should meet the needs of the end-users (clinicians and patients) in a specific context. On the basis of that, different types of transducers (electrochemical, optical, piezoelectric, *etc.*), platforms (paper, plastic, silicon dioxide, *etc.*), fabrication technologies (microfabrication in clean room, printing, laser scribing, *etc.*), and bioreceptors types (antibodies, aptamers, enzymes, *etc.*) can be selected and explored. The output of this process is the definition of the type of diagnostic device required, spacing from a low-cost disposable naked-eye device (such as an LFA) to a reusable precision benchtop system (such as a fluorescence, interferometric, or SPR test). Both the definition of the problem and of the user needs with the appropriate choice of all the future diagnostic device components strongly benefit from interdisciplinary teams and collaborative environments, being a key point for a good diagnostic device conception and design.

### Laboratory Testing

In practice, the first step in developing a diagnostic device is to demonstrate its feasibility or its ability to detect the target analyte in ideal conditions, such as the pure analyte in a buffer solution and in commercially available biological fluids (artificial saliva and urine, serum, blood, *etc*.). This proof-of-concept system is usually bulky and laboratory-based, working only in a controlled environment and under very specific conditions. Nevertheless, this stage for a biosensor is crucial, and its related work is often under-appreciated: work in this stage is rarely covered by funding through public project calls and rarely published in leading journals unless the underlying technology is extremely innovative. The link between innovation and the social utility of the related diagnostic test is not always evident. Many of the technologies published in high-impact journals never reach the market, and a few, marginally innovative technologies are broadly used in the clinics having a strong impact on the life of millions of patients. If the proof-of-concept system is working, the following phase consists in the determination of analytical metrics such as analytical sensitivity, the limit of detection (LoD), analytical selectivity, trueness, and precision of these devices in biological fluids.^36^ Extensive and time-consuming studies are performed to define the best combination of a system’s parameters to achieve a sufficiently high performance for its specific application. This is typically the stage at which patenting is considered and assessed.

### Clinical Trials

After the device has been successfully characterized and tested in a laboratory setting, the next step is its clinical validation using real clinical samples, in the conditions of the intended use. Even clinical entities sometimes have difficulties to find clinically relevant specimens, depending on the type of biological sample to be collected, required storage conditions, and the number of patients related to a particular type of infection. Ebola samples, for example, are difficult to find, since there are only few cases in the world, but even influenza samples have become difficult to find, since it became very rare after the COVID-19 pandemic. For a nonclinical entity, things are even more complicated. The three main obstacles that nonclinical entities must face are (1) the inadequacy of the developer infrastructure to deal with patient samples with a level of biosafety (BSL) above 1, which requires dedicated areas and facilities,^55^ (2) the ethical issues to obtain and work with such samples and the ethical committee approval related costs,^66,67^ and (3) the unavoidable worsening of the analytical performance of the test moving from in vitro conditions to the complexity and patient-to-patient variability of body fluid samples. Collaboration with clinical partners is the best solution to the first two obstacles, using appropriate spaces and harnessing ongoing or past clinical trials samples, already approved for research purposes. Alternatively, it is possible to purchase validated samples from companies, which takes care of both the sample collection and the ethical aspects, with the related costs in terms of money and time. The last critical obstacle is to avoid sample preprocessing steps, if possible. The technologies able to avoid it typically required many years of R&D to reach that goal.

### Regulatory Review

Regulatory agencies evaluate *in vitro* diagnostics (IVDs) and medical devices. Each country has its regulatory agency that should ensure that all the IVDs available on the market have been adequately assessed for analytical and clinical validity.^68^ Additionally, the regulatory agencies contribute to the control of the IVDs in the premarket step and in the postmarket surveillance, to ensure that the IVDs that are used remain safe and effective for the final users.^69^ In Europe, most IVDs can still be placed on the market by their manufacturers solely based on an EC Declaration of Conformity (known as “Self-Certification”).^70^ This applies to all CE-marked IVD kits for COVID-19 testing, regardless of their underlying technology and operating principle and of their use environment.^71^ This current lack of regulatory oversight for IVDs is even more striking, as these kits will be considered of the highest-risk class under the new Regulation EU/2017/746 for IVDs (IVDR) and therefore subject to a stringent conformity assessment procedure for CE certification, with involvement of third parties, including Notified Bodies and EU Reference Laboratories.^72^ The IVDR will be applicable in one year (May 26th, 2022), and in the meantime several initiatives have been launched to provide a minimum assurance of reliability for such tests.

In the United States, a temporary Emergency Use Authorization (EUA) from the Food and Drug Administration (FDA) can be sought by manufacturers and laboratories for IVDs for the detection and/or diagnosis of SARS-CoV-2. Unlike the more restrictive EU approach, developers of tests based on other technologies are encouraged to discuss with the FDA the most suitable approach to obtain the corresponding EUA. In addition, in the U.S. the so-called laboratory-developed tests (or LDT) are much more widespread and accepted than the in-house test concept in the EU, and the FDA considers them appropriate for COVID-19 testing when based on molecular assays and performed in accredited, Clinical Laboratory Improvement Amendments (CLIA)-certified laboratories.^73,74^

### Scaleup and Market Launch

Although scaleup might be the last item on the agenda, plans to bring the technology to market should be considered and well-defined from the initial stages. It is critical to consider from the beginning the scalability of the proposed fabrication and functionalization technology. In this way, the scale-up phase time could be drastically reduced, producing a market-winning product. As scientists, the know-how of planning a scale-up is very daunting, so usually this is outsourced to pilot plants, or ad-hoc spin-offs are created with this purpose. These partners will work closely with the (scientific) development team to ensure that the design protocols and resources adhere to manufacturing principles (Good Manufacturing Practices (GMP))^75^ and that the target quantities can meet the anticipated volumes for the pilot launch. Furthermore, having a comprehensive, yet adaptable, quality assurance/control (QA/QC) plan is very important for the scaleup. This mitigates any unforeseeable situations, that is, amounts or costs of resources, and ensures that the pilot launch will not be delayed.

## The Bottlenecks

Efficient diagnostic devices are one of the most sought-after elements in a pandemic situation, as seen in the ongoing COVID-19 case.^76^ Given the surge in demand, it is of utmost importance to do a thorough study of the process of development and production of diagnostic tests in order to elucidate its bottlenecks, both in terms of time and funding. Each stage of the process is characterized by limitations related to the different stakeholders involved. These stakeholders include patients, clinicians, researchers, companies, investors, and policy makers.^77^

### Bottlenecks in Research Stages

A major bottleneck in the early stages of the development of diagnostic tests in pandemic situations is the availability of information regarding the etiological agent (*i.e.*, the agent causing the disease). Indeed, it is not possible to develop a diagnostic device without knowing either the etiological agent’s genome^78^ or antigens^79^ or the elicited immunological response.^80^ Additionally, access to materials and supplies necessary for the regular development of research can be compromised if the supply chain is interrupted either by the severity of the pandemic or because of the policies followed to halt its expansion.^81^

In the conception and design phase it is fundamental to define the type of biological sample that is going to be analyzed, the clinical range that needs to be reached, the techniques or molecules that are going to be used to perform the detection, the platform used, the user’s needs, *etc*. The initial idea of the device needs to be carefully described, justified, and classified according to the corresponding regulatory entity rules in order to, eventually, expedite the process of approval of the device.^82^ Any difficulty to have access to this information can potentially cause a significant delay of the overall process. Even worse, if this information is imprecise or wrong the process could be conducted toward a wrong direction resulting in the complete failure of the development process of the diagnostic test.

Prototyping, or preclinical research stage, also entails certain bottlenecks. It is during this stage that users’ expectations and usability as well as a future scaleup of the product should be addressed to avoid future redesigns of the device, which may cause important delays due to unnecessary iterations and extra costs. Appearance and user-friendliness of the device, supply chain limitations, and a simple and optimal industrial fabrication guide all need to be considered. Experience in similar endeavors by researchers and engineers is key to address all these possible sources of future complications sooner rather than later, minimizing the unnecessary iterations. In this regard, a smooth interdisciplinary communication between researchers and clinicians is necessary to approach the final users’ preferences, and communication between researchers and the industry is important to avoid setbacks.^83^

Clinical trials with the developed diagnostic tests involve testing real samples, whether directly from patients or using samples collected by a healthcare center. In this and the previous phase, the highest obstacle usually is specificity (*i.e.*, interference by other molecules in the sample of interest and variability of their concentrations in samples collected in different moments from different individuals). Facing this obstacle can lead to a severe prolongation of the development period. After the clinical trials, diagnostic tests undergo scrutiny from the pertinent regulatory authority. For a fluent process, all requirements from the authority should have been accounted for in all previous stages before presenting the device.^84^

### Bottlenecks in Market Stages

Once a diagnostic test enters the market, a lot of work is still required to improve the device’s performance and guarantee its success. User feedback, particularly from clinical personnel, and refinement are essential for improving the accuracy of the diagnostic device. Over- or under-confidence on a diagnostic test can lead to a misdiagnosis and its undesirable side effects.^85^ Aside from lab-tested and validated performance aspects, such as accuracy, repeatability, sensitivity, and meeting clinical requirements, the translation of a POC technology to a large-scale production is not always as straightforward as it seems. Planning for the success of a diagnostic product also includes an understanding of the demands of production and extensive market research. For example, unlike in lab settings, mass production can mean the consumption of materials and reagents 100 times faster with rigid and unforgiving deadlines. Moreover, the adaptability of production should also be considered especially in terms of variability between batches of raw material—will a change part way through affect the device performance? Although in theory, all batches should be the same and be of high quality and purity, in practice, this does not happen in all the cases. Assay performances may also vary batch-to-batch, as many biological reagents are sold in terms of purity without an assessment of their reactivity. Mass production is also a critical point for diagnostic tests involving nanomaterials. The large-scale production of some nanomaterials still poses a challenge with respect to controlling the size, defects, and stability of the nanomaterials during a large-scale production. In addition, the synthesis methods should ideally be low-cost. Protocols that integrate the nanomaterials into the diagnostic devices during a large-scale production must preserve the outstanding properties of the nanomaterials. Another challenge is the biofunctionalization of nanomaterials at scale and with high reproducibility. The shelf life and storage should be assessed early on and can be critical for POC diagnostics including biological reagents and materials (polymeric housing, metallic electrodes, adhesives). Exposure to heat or moisture can degrade both materials and reagents.

### Regulatory Bottlenecks

The regulatory aspects have a central role in the introduction of a POC device. In the case of the current pandemic, the WHO has established the Emergency Use Listing (EUL) procedure for IVDs to detect SARS-CoV-2 in order to determine their eligibility through a procurement process by the WHO or its partners. In this procedure, data pertaining to the quality, safety, and performance of IVDs are critically assessed by the WHO, and upon review a recommendation for EUL to the assessed product is eventually granted.^78,86^ The European Commission recently published a working document to establish proposed performance criteria for COVID-19 tests, which includes molecular- and immunologic-based methods. Despite this effort, such a document highlights the difficulty in establishing a clear link between validation data and specific commercial devices or in defining the reliability of the identified performance data, often not validated by third parties.^87^ Beyond performance criteria, other aspects may be critical for the certification of the devices, especially if intended to be developed as tests for lay users (known as “self-tests”).

When it comes to the development of IVDs based on nanomaterials, regulatory aspects become even more challenging. A representative class of nanomaterials that is explored in academic research for the development of IVDs still does not have its biosafety level fully understood and its use regulated. Even though there have been some advances, such as the publication of ISO/TR 10993-22:2017,^88^ which describes considerations for the biological evaluation of medical devices that are composed of or contain nanomaterials, we still have a lack of international regulatory guidelines for evaluating the safety of different types of nanomaterials integrated into IVDs.

### Privacy Issues

The widespread usage of mobile technologies generates clear synergies with diagnostic tests, but it also brings ethical issues concerning the privacy of patients.^89,90^ Coupling diagnostic devices to contact-tracing apps might not receive trust from users who may fear that the collected data regarding their location might be used for non-COVID-19 related purposes. Many questions must be answered in order to understand to what extent contact-tracing apps are ethically justifiable.^91^ Although the purpose of surveillance testing is not ultimately that of returning a diagnostic result to an individual but to obtain information at a population level,^92^ public authorities must remain aware that trust from citizens is earned but not enforced. This is common in different organizational environments.^93^ In this regard, it is possible to implement nonmandatory contact-tracing apps that preserve privacy, thus balancing the needs and goals of users, policy makers, and developers. Ideally, this should be the case when the values of each stakeholder are aligned.^94^ Moreover, a transparent and clear communication with the user is fundamental to gain their trust and successfully implement the adoption of these advantageous technologies.

## Improvements for Facing Future Pandemics

There is no doubt that high-performance and reliable devices need high-quality research efforts and time for their development. However, on the basis of our analysis of the bottlenecks during the development process, and from our direct experience in the preclinical and clinical stages, we have suggestions for the minimization of unnecessary time loss and for the improvement of the preparedness for the next pandemics.

The first concept we think should be explored is the “a test for each purpose” paradigm. Currently, we pursue the development of a sensor with very high performance/manufacturability and low cost to be used as a valid alternative to the gold standard molecular testing. As we have seen, this takes time, but more importantly, that performance may not be needed for every testing purpose.

Models of the infection spreading could in fact give a “score” to different places and events on the basis of their frequency, prevalence in the area, and of the potential consequences of a false positive, for example, with a color scale. Accordingly, different diagnostic tests designed for that color code may be employed, each with an optimized performance/cost/response time for the respective color code. It is evident that testing in an airport before an international flight would need a higher sensitivity with respect to a daily test in a small school.

To make this paradigm possible and the development process lean and faster, we think that the consolidation of a reduced set of technologies for the rapid targeting, monitoring, and tracking of new pathogens should be prioritized. The concept is to have an “outbreak nanodiagnostic survival kit”, that is, a minimum set of already-verified materials, techniques, and methods for the fast production and scaling of a first screening tool. Many nanomaterials and nanotechnological methods are good candidates to be included in the kit due to the astonishing properties they confer to the diagnostic devices, but it is fundamental to set up routes for their scale-up. As we have seen, critical data about new pathogens can be obtained and communicated in fast times; thus, such a kit would ensure its rapid and proper use minimizing the development process risks and times.

Driving the scientific community in these directions may be easier if the implementation research and advancements at the highest TRLs may be better considered by high-impact journals, encouraging them.

Another issue that could be faced is the one related to statistical illiteracy. Most of the people are not confident about the implications and effects of different sensitivity and accuracy values that characterize the performance of each diagnostic test. First, these should be certified by independent entities and available to the user (*e.g*., on a public downloadable database, such as the FIND one). Second, their meaning should be made clear for everybody and enter in the common culture.

In all these approaches to the future pandemics a transversal factor, which makes the difference, is interdisciplinary research teams and tight collaborations between all the actors involved in the development process. The collaboration of these actors as a unique unit and not as a chain of independent black boxes would allow a drastic optimization of the time and effort needed for the development of a nanodiagnostic device.

Finally, the last innovation we envision is the establishment of international biobanks containing verified patient samples. Having access to those samples could be either related to specific grants or by an employment of strict criteria, such as proven high-enough TRLs. Potentially the biobanks may also provide laboratory spaces with BSL 2 and 3, which are generally difficult to find and even more difficult to get access into. This would allow research groups even with limited collaborations with clinicians to get access to readily available samples and facilities to perform the development of diagnostic devices. We understand this is far from being simple and straightforward, but we believe it is worth pursuing.

## Vocabulary

SARS-CoV-2: The acronym given to the severe acute respiratory syndrome coronavirus 2, causing the coronavirus disease (COVID-19)
Nanodiagnostics: A term intended to indicate diagnostic devices that include a nanotechnological component (*e.g.*, nanoparticles, 2D nanomaterials, quantum dots, etc.) to perform the detection. Most often the nanocomponent increases the sensitivity with respect to standard diagnostic devices
Molecular test: This refers to a PCR-based test to diagnose COVID-19 by analyzing the RNA present in an oral sample of the patient
Antigenic test: This refers to a diagnostic test to analyze an oral sample detecting the presence of the antigens (proteins) of the SARS-CoV-2 virus, typically through a protein-antibody interaction
Serological test: This refers to a diagnostic test for the detection of specific anti-SARS-CoV-2 antibodies in a blood sample of the patient, with the aim to check for his/her immunization against SARS-CoV-2
TRL: This stands for Technology Readiness Level, and it is a score to define the level of advancement of a new technological product/idea. It is used to classify research projects, starting levels, and advancements but also business ideas and plans.

## References

[ref1] Wang H.; Li X.; Li T.; Zhang S.; Wang L.; Wu X.; Liu J. The Genetic Sequence, Origin, and Diagnosis of SARS-CoV-2. Eur. J. Clin. Microbiol. Infect. Dis. 2020, 39 (9), 1629–1635. 10.1007/s10096-020-03899-4. 32333222 PMC7180649

[ref2] Chang D.; Lin M.; Wei L.; Xie L.; Zhu G.; Dela Cruz C. S.; Sharma L. Epidemiologic and Clinical Characteristics of Novel Coronavirus Infections Involving 13 Patients Outside Wuhan, China. JAMA 2020, 323 (11), 1092–1093. 10.1001/jama.2020.1623. 32031568 PMC7042871

[ref3] Li D.; Jin M.; Bao P.; Zhao W.; Zhang S. Clinical Characteristics and Results of Semen Tests among Men with Coronavirus Disease 2019. JAMA Netw. Open 2020, 3 (5), e208292 10.1001/jamanetworkopen.2020.8292. 32379329 PMC7206502

[ref4] Holshue M. L.; DeBolt C.; Lindquist S.; Lofy K. H.; Wiesman J.; Bruce H.; Spitters C.; Ericson K.; Wilkerson S.; Tural A.; Diaz G.; Cohn A.; Fox L.; Patel A.; Gerber S. I.; Kim L.; Tong S.; Lu X.; Lindstrom S.; Pallansch M. A.; et al. First Case of 2019 Novel Coronavirus in the United States. N. Engl. J. Med. 2020, 382 (10), 929–936. 10.1056/NEJMoa2001191. 32004427 PMC7092802

[ref5] Astuti I.; Ysrafil Severe Acute Respiratory Syndrome Coronavirus 2 (SARS-CoV-2): An Overview of Viral Structure and Host Response. Diabetes Metab. Syndr. Clin. Res. Rev. 2020, 14 (4), 407–412. 10.1016/j.dsx.2020.04.020. PMC7165108 32335367

[ref6] Liu Y.; Rocklöv J. The Reproductive Number of the Delta Variant of SARS-CoV-2 Is Far Higher Compared to the Ancestral SARS-CoV-2 Virus. J. Travel Med. 2021. Article ASAP. 10.1093/jtm/taab124. PMC8436367 34369565

[ref7] Burki T. K. Lifting of COVID-19 Restrictions in the UK and the Delta Variant. Lancet Respir. Med. 2021, 9 (8), e85 10.1016/S2213-2600(21)00328-3. 34265238 PMC8275031

[ref8] Buonaguro L.; Tagliamonte M.; Tornesello M. L.; Buonaguro F. M. SARS-CoV-2 RNA Polymerase as Target for Antiviral Therapy. J. Transl. Med. 2020, 18 (1), 185 10.1186/s12967-020-02355-3. 32370758 PMC7200052

[ref9] Petrosillo N.; Viceconte G.; Ergonul O.; Ippolito G.; Petersen E. COVID-19, SARS and MERS: Are They Closely Related?. Clin. Microbiol. Infect. 2020, 26 (6), 729–734. 10.1016/j.cmi.2020.03.026. 32234451 PMC7176926

[ref10] Ou X.; Liu Y.; Lei X.; Li P.; Mi D.; Ren L.; Guo L.; Guo R.; Chen T.; Hu J.; Xiang Z.; Mu Z.; Chen X.; Chen J.; Hu K.; Jin Q.; Wang J.; Qian Z. Characterization of Spike Glycoprotein of SARS-CoV-2 on Virus Entry and Its Immune Cross-Reactivity with SARS-CoV. Nat. Commun. 2020, 11 (1), 1620 10.1038/s41467-020-15562-9. 32221306 PMC7100515

[ref11] Walls A. C.; Park Y.-J.; Tortorici M. A.; Wall A.; McGuire A. T.; Veesler D. Structure, Function, and Antigenicity of the SARS-CoV-2 Spike Glycoprotein. Cell 2020, 181 (2), 281–292. 10.1016/j.cell.2020.02.058. 32155444 PMC7102599

[ref12] Tilocca B.; Soggiu A.; Sanguinetti M.; Babini G.; De Maio F.; Britti D.; Zecconi A.; Bonizzi L.; Urbani A.; Roncada P. Immunoinformatic Analysis of the SARS-CoV-2 Envelope Protein as a Strategy to Assess Cross-Protection against COVID-19. Microbes Infect. 2020, 22 (4–5), 182–187. 10.1016/j.micinf.2020.05.013. 32446902 PMC7241347

[ref13] Yin C. Genotyping Coronavirus SARS-CoV-2: Methods and Implications. Genomics 2020, 112 (5), 3588–3596. 10.1016/j.ygeno.2020.04.016. 32353474 PMC7184998

[ref14] Li Q.; Guan X.; Wu P.; Wang X.; Zhou L.; Tong Y.; Ren R.; Leung K. S. M.; Lau E. H. Y.; Wong J. Y.; Xing X.; Xiang N.; Wu Y.; Li C.; Chen Q.; Li D.; Liu T.; Zhao J.; Liu M.; Tu W.; et al. Early Transmission Dynamics in Wuhan, China, of Novel Coronavirus-Infected Pneumonia. N. Engl. J. Med. 2020, 382 (13), 1199–1207. 10.1056/NEJMoa2001316. 31995857 PMC7121484

[ref15] Andersen K. G.; Rambaut A.; Lipkin W. I.; Holmes E. C.; Garry R. F. The Proximal Origin of SARS-CoV-2. Nat. Med. 2020, 26 (4), 450–452. 10.1038/s41591-020-0820-9. 32284615 PMC7095063

[ref16] St John A.; Price C. P. Existing and Emerging Technologies for Point-of-Care Testing. Clin. Biochem. Rev. 2014, 35 (3), 155–167. 25336761 PMC4204237

[ref17] Gervais L.; de Rooij N.; Delamarche E. Microfluidic Diagnostic Devices: Microfluidic Chips for Point-of-Care Immunodiagnostics. Adv. Mater. 2011, 23 (24), H208–H208. 10.1002/adma.201190098. 21567479

[ref18] Delamarche E.; Bernard A.; Schmid H.; Michel B.; Biebuyck H. Patterned Delivery of Immunoglobulins to Surfaces Using Microfluidic Networks. Science 1997, 276 (5313), 779–781. 10.1126/science.276.5313.779. 9115199

[ref19] Guangzhou Wondfo Biotech Co. LTD. COVID-19 - Wondfo https://en.wondfo.com.cn/es/covid-19-5/ (accessed 2020-04-28).

[ref20] Crozier A.; Rajan S.; Buchan I.; McKee M. Put to the Test: Use of Rapid Testing Technologies for Covid-19. BMJ. 2021, 372, n208 10.1136/bmj.n208. 33536228

[ref21] Niemz A.; Ferguson T. M.; Boyle D. S. Point-of-Care Nucleic Acid Testing for Infectious Diseases. Trends Biotechnol. 2011, 29 (5), 240–250. 10.1016/j.tibtech.2011.01.007. 21377748 PMC3746968

[ref22] Nichols J. H. Reducing Medical Errors at the Point of Care. Lab. Med. 2005, 36 (5), 275–277. 10.1309/NXXWJ31PWFHT7L1Q.

[ref23] Shaw J. L. V. Practical Challenges Related to Point of Care Testing. Pract. Lab. Med. 2016, 4, 22–29. 10.1016/j.plabm.2015.12.002. 28856189 PMC5574506

[ref24] Kumar A. A.; Hennek J. W.; Smith B. S.; Kumar S.; Beattie P.; Jain S.; Rolland J. P.; Stossel T. P.; Chunda-Liyoka C.; Whitesides G. M. From the Bench to the Field in Low-Cost Diagnostics: Two Case Studies. Angew. Chem., Int. Ed. 2015, 54 (20), 5836–5853. 10.1002/anie.201411741. 25914299

[ref25] Sachdeva S.; Davis R. W.; Saha A. K. Microfluidic Point-of-Care Testing: Commercial Landscape and Future Directions. Front. Bioeng. Biotechnol. 2021, 8, 1537 10.3389/fbioe.2020.602659. PMC7843572 33520958

[ref26] Kelley S. O. COVID-19: A Crisis Creating New Opportunities for Sensing. ACS Sensors 2021, 6 (4), 1407–1407. 10.1021/acssensors.1c00687. 33887917

[ref27] Yousefi H.; Mahmud A.; Chang D.; Das J.; Gomis S.; Chen J. B.; Wang H.; Been T.; Yip L.; Coomes E.; Li Z.; Mubareka S.; McGeer A.; Christie N.; Gray-Owen S.; Cochrane A.; Rini J. M.; Sargent E. H.; Kelley S. O. Detection of SARS-CoV-2 Viral Particles Using Direct, Reagent-Free Electrochemical Sensing. J. Am. Chem. Soc. 2021, 143 (4), 1722–1727. 10.1021/jacs.0c10810. 33481575

[ref28] Wu F.; Zhao S.; Yu B.; Chen Y.-M.; Wang W.; Song Z.-G.; Hu Y.; Tao Z.-W.; Tian J.-H.; Pei Y.-Y.; Yuan M.-L.; Zhang Y.-L.; Dai F.-H.; Liu Y.; Wang Q.-M.; Zheng J.-J.; Xu L.; Holmes E. C.; Zhang Y.-Z. A New Coronavirus Associated with Human Respiratory Disease in China. Nature 2020, 579 (7798), 265–269. 10.1038/s41586-020-2008-3. 32015508 PMC7094943

[ref29] Zhou P.; Yang X.-L.; Wang X.-G.; Hu B.; Zhang L.; Zhang W.; Si H.-R.; Zhu Y.; Li B.; Huang C.-L.; Chen H.-D.; Chen J.; Luo Y.; Guo H.; Jiang R.-D.; Liu M.-Q.; Chen Y.; Shen X.-R.; Wang X.; Zheng X.-S.; et al. A Pneumonia Outbreak Associated with a New Coronavirus of Probable Bat Origin. Nature 2020, 579 (7798), 270–273. 10.1038/s41586-020-2012-7. 32015507 PMC7095418

[ref30] SARS-CoV-2 Molecular Assay Evaluation: Results. https://www.finddx.org/sarscov2-eval-molecular/molecular-eval-results/ (accessed 2020-08-01).

[ref31] Deeks J. J.; Dinnes J.; Takwoingi Y.; Davenport C.; Leeflang M. M. G.; Spijker R.; Hooft L.; Van den Bruel A.; Emperador D.; Dittrich S. Diagnosis of SARS-CoV-2 Infection and COVID-19: Accuracy of Signs and Symptoms; Molecular, Antigen, and Antibody Tests; and Routine Laboratory Markers. Cochrane Database Syst. Rev. 2020, (4), 1–14. 10.1002/14651858.CD013596.

[ref32] Carter L. J.; Garner L. V.; Smoot J. W.; Li Y.; Zhou Q.; Saveson C. J.; Sasso J. M.; Gregg A. C.; Soares D. J.; Beskid T. R.; Jervey S. R.; Liu C. Assay Techniques and Test Development for COVID-19 Diagnosis. ACS Cent. Sci. 2020, 6 (5), 591–605. 10.1021/acscentsci.0c00501. 32382657 PMC7197457

[ref33] Dortet L.; Emeraud C.; Vauloup-Fellous C.; Khecharem M.; Ronat J.-B.; Fortineau N.; Roque-Afonso A.-M.; Naas T. Rapid Determination of SARS-CoV-2 Antibodies Using a Bedside, Point-of-Care, Serological Test. Emerging Microbes Infect. 2020, 9 (1), 2212–2221. 10.1080/22221751.2020.1826892. PMC7580567 32969769

[ref34] Mercer T. R.; Salit M. Testing at Scale during the COVID-19 Pandemic. Nat. Rev. Genet. 2021, 22, 415 10.1038/s41576-021-00360-w. 33948037 PMC8094986

[ref35] Parikh R.; Mathai A.; Parikh S.; Chandra Sekhar G.; Thomas R. Understanding and Using Sensitivity, Specificity and Predictive Values. Indian J. Ophthalmol. 2008, 56 (1), 45 10.4103/0301-4738.37595. 18158403 PMC2636062

[ref36] Borysiak M. D.; Thompson M. J.; Posner J. D. Translating Diagnostic Assays from the Laboratory to the Clinic: Analytical and Clinical Metrics for Device Development and Evaluation. Lab Chip 2016, 16 (8), 1293–1313. 10.1039/C6LC00015K. 27043204

[ref37] FIND. Foundation for Innovative New Diagnostics - Test Directory. https://www.finddx.org/test-directory/ (accessed 2021-05-05).

[ref38] Land K. J.; Boeras D. I.; Chen X.-S.; Ramsay A. R.; Peeling R. W. REASSURED Diagnostics to Inform Disease Control Strategies, Strengthen Health Systems and Improve Patient Outcomes. Nat. Microbiol. 2019, 4 (1), 46–54. 10.1038/s41564-018-0295-3. 30546093 PMC7097043

[ref39] Nam J.-M.; et al. Nanoparticle-Based Bio-Bar Codes for the Ultrasensitive Detection of Proteins. Science 2003, 301 (5641), 1884–1886. 10.1126/science.1088755. 14512622

[ref40] Walt D. R. CHEMISTRY: Miniature Analytical Methods for Medical Diagnostics. Science 2005, 308 (5719), 217–219. 10.1126/science.1108161. 15821081

[ref41] Pérez-López B.; Merkoçi A. Nanomaterials Based Biosensors for Food Analysis Applications. Trends Food Sci. Technol. 2011, 22 (11), 625–639. 10.1016/j.tifs.2011.04.001.

[ref42] Weiss C.; Carriere M.; Fusco L.; Capua I.; Regla-Nava J. A.; Pasquali M.; Scott J. A.; Vitale F.; Unal M. A.; Mattevi C.; Bedognetti D.; Merkoçi A.; Tasciotti E.; Yilmazer A.; Gogotsi Yu; Stellacci F.; Delogu L. G. Toward Nanotechnology-Enabled Approaches against the COVID-19 Pandemic. ACS Nano 2020, 14 (6), 6383–6406. 10.1021/acsnano.0c03697. 32519842

[ref43] Parolo C.; Sena-Torralba A.; Bergua J. F.; Calucho E.; Fuentes-Chust C.; Hu L.; Rivas L.; Álvarez-Diduk R.; Nguyen E. P.; Cinti S.; Quesada-González D.; Merkoçi A. Tutorial: Design and Fabrication of Nanoparticle-Based Lateral-Flow Immunoassays. Nat. Protoc. 2020, 15 (12), 3788–3816. 10.1038/s41596-020-0357-x. 33097926

[ref44] Kurbanoglu S.; Ozkan S. A.; Merkoçi A. Nanomaterials-Based Enzyme Electrochemical Biosensors Operating through Inhibition for Biosensing Applications. Biosens. Bioelectron. 2017, 89, 886–898. 10.1016/j.bios.2016.09.102. 27818056

[ref45] Morales-Narváez E.; Merkoçi A. Graphene Oxide as an Optical Biosensing Platform. Adv. Mater. 2012, 24 (25), 3298–3308. 10.1002/adma.201200373. 22628274

[ref46] Mokhtarzadeh A.; Eivazzadeh-Keihan R.; Pashazadeh P.; Hejazi M.; Gharaatifar N.; Hasanzadeh M.; Baradaran B.; de la Guardia M. Nanomaterial-Based Biosensors for Detection of Pathogenic Virus. TrAC, Trends Anal. Chem. 2017, 97, 445–457. 10.1016/j.trac.2017.10.005. PMC7126209 32287543

[ref47] Morales-Narváez E.; Baptista-Pires L.; Zamora-Gálvez A.; Merkoçi A. Graphene-Based Biosensors: Going Simple. Adv. Mater. 2017, 29, 1604905 10.1002/adma.201604905. 27896856

[ref48] Nguyen E. P.; de Carvalho Castro Silva C.; Merkoçi A. Recent Advancement in Biomedical Applications on the Surface of Two-Dimensional Materials: From Biosensing to Tissue Engineering. Nanoscale 2020, 12 (37), 19043–19067. 10.1039/D0NR05287F. 32960195

[ref49] Nakatsuka N.; Yang K.-A.; Abendroth J. M.; Cheung K. M.; Xu X.; Yang H.; Zhao C.; Zhu B.; Rim Y. S.; Yang Y.; Weiss P. S.; Stojanovic M. N.; Andrews A. M. Aptamer-Field-Effect Transistors Overcome Debye Length Limitations for Small-Molecule Sensing. Science 2018, 362 (6412), 319–324. 10.1126/science.aao6750. 30190311 PMC6663484

[ref50] Roche E. T. A Protein Sandwich Enables Real-Time *in Vivo* Biomarker Measurement. Sci. Transl. Med. 2021, 13 (575), eabg1758 10.1126/scitranslmed.abg1758.

[ref51] Idili A.; Parolo C.; Alvarez-Diduk R.; Merkoçi A. Rapid and Efficient Detection of the SARS-CoV-2 Spike Protein Using an Electrochemical Aptamer-Based Sensor. ACS Sensors 2021, 6 (8), 3093–3101. 10.1021/acssensors.1c01222. 34375076 PMC8370117

[ref52] Alafeef M.; Dighe K.; Moitra P.; Pan D. Rapid, Ultrasensitive, and Quantitative Detection of SARS-CoV-2 Using Antisense Oligonucleotides Directed Electrochemical Biosensor Chip. ACS Nano 2020, 14 (12), 17028–17045. 10.1021/acsnano.0c06392. 33079516

[ref53] Baker A. N.; Richards S.-J.; Guy C. S.; Congdon T. R.; Hasan M.; Zwetsloot A. J.; Gallo A.; Lewandowski J. R.; Stansfeld P. J.; Straube A.; Walker M.; Chessa S.; Pergolizzi G.; Dedola S.; Field R. A.; Gibson M. I. The SARS-COV-2 Spike Protein Binds Sialic Acids and Enables Rapid Detection in a Lateral Flow Point of Care Diagnostic Device. ACS Cent. Sci. 2020, 6 (11), 2046–2052. 10.1021/acscentsci.0c00855. 33269329 PMC7523238

[ref54] Lin Q.; Wen D.; Wu J.; Liu L.; Wu W.; Fang X.; Kong J. Microfluidic Immunoassays for Sensitive and Simultaneous Detection of IgG/IgM/Antigen of SARS-CoV-2 within 15 min. Anal. Chem. 2020, 92 (14), 9454–9458. 10.1021/acs.analchem.0c01635. 32615038

[ref55] Mavrikou S.; Moschopoulou G.; Tsekouras V.; Kintzios S. Development of a Portable, Ultra-Rapid and Ultra-Sensitive Cell-Based Biosensor for the Direct Detection of the SARS-CoV-2 S1 Spike Protein Antigen. Sensors 2020, 20 (11), 3121 10.3390/s20113121. PMC7309076 32486477

[ref56] Moitra P.; Alafeef M.; Dighe K.; Frieman M. B.; Pan D. Selective Naked-Eye Detection of SARS-CoV-2 Mediated by N Gene Targeted Antisense Oligonucleotide Capped Plasmonic Nanoparticles. ACS Nano 2020, 14 (6), 7617–7627. 10.1021/acsnano.0c03822. 32437124

[ref57] Qiu G.; Gai Z.; Tao Y.; Schmitt J.; Kullak-Ublick G. A.; Wang J. Dual-Functional Plasmonic Photothermal Biosensors for Highly Accurate Severe Acute Respiratory Syndrome Coronavirus 2 Detection. ACS Nano 2020, 14 (5), 5268–5277. 10.1021/acsnano.0c02439. 32281785

[ref58] Seo G.; Lee G.; Kim M. J.; Baek S.-H.; Choi M.; Ku K. B.; Lee C.-S.; Jun S.; Park D.; Kim H. G.; Kim S.-J.; Lee J.-O.; Kim B. T.; Park E. C.; Kim S. I. Rapid Detection of COVID-19 Causative Virus (SARS-CoV-2) in Human Nasopharyngeal Swab Specimens Using Field-Effect Transistor-Based Biosensor. ACS Nano 2020, 14 (4), 5135–5142. 10.1021/acsnano.0c02823. 32293168

[ref59] Shao W.; Shurin M. R.; Wheeler S. E.; He X.; Star A. Rapid Detection of SARS-CoV-2 Antigens Using High-Purity Semiconducting Single-Walled Carbon Nanotube-Based Field-Effect Transistors. ACS Appl. Mater. Interfaces 2021, 13 (8), 10321–10327. 10.1021/acsami.0c22589. 33596036

[ref60] Torrente-Rodríguez R. M.; Lukas H.; Tu J.; Min J.; Yang Y.; Xu C.; Rossiter H. B.; Gao W. SARS-CoV-2 RapidPlex: A Graphene-Based Multiplexed Telemedicine Platform for Rapid and Low-Cost COVID-19 Diagnosis and Monitoring. Matter 2020, 3 (6), 1981–1998. 10.1016/j.matt.2020.09.027. 33043291 PMC7535803

[ref61] Zhao H.; Liu F.; Xie W.; Zhou T.-C.; OuYang J.; Jin L.; Li H.; Zhao C.-Y.; Zhang L.; Wei J.; Zhang Y.-P.; Li C.-P. Ultrasensitive Supersandwich-Type Electrochemical Sensor for SARS-CoV-2 from the Infected COVID-19 Patients Using a Smartphone. Sens. Actuators, B 2021, 327, 128899 10.1016/j.snb.2020.128899. PMC7489230 32952300

[ref62] Zhu X.; Wang X.; Han L.; Chen T.; Wang L.; Li H.; Li S.; He L.; Fu X.; Chen S.; Xing M.; Chen H.; Wang Y. Multiplex Reverse Transcription Loop-Mediated Isothermal Amplification Combined with Nanoparticle-Based Lateral Flow Biosensor for the Diagnosis of COVID-19. Biosens. Bioelectron. 2020, 166, 112437 10.1016/j.bios.2020.112437. 32692666 PMC7361114

[ref63] Shan B.; Broza Y. Y.; Li W.; Wang Y.; Wu S.; Liu Z.; Wang J.; Gui S.; Wang L.; Zhang Z.; Liu W.; Zhou S.; Jin W.; Zhang Q.; Hu D.; Lin L.; Zhang Q.; Li W.; Wang J.; Liu H.; et al. Multiplexed Nanomaterial-Based Sensor Array for Detection of COVID-19 in Exhaled Breath. ACS Nano 2020, 14 (9), 12125–12132. 10.1021/acsnano.0c05657. 32808759

[ref64] Ding X.; Yin K.; Li Z.; Lalla R. V.; Ballesteros E.; Sfeir M. M.; Liu C. Ultrasensitive and Visual Detection of SARS-CoV-2 Using All-in-One Dual CRISPR-Cas12a Assay. Nat. Commun. 2020, 11 (1), 4711 10.1038/s41467-020-18575-6. 32948757 PMC7501862

[ref65] Sadana A. Market Size and Economics for Biosensors. In Fractal Binding and Dissociation Kinetics for Different Biosensor Applications*;* Sadana A., Ed.; Elsevier: Amsterdam, The Netherlands, 2005; pp 265–299. 10.1016/B978-044451945-0/50014-5.

[ref66] Altawalbeh S. M.; Alkhateeb F. M.; Attarabeen O. F. Ethical Issues in Consenting Older Adults: Academic Researchers and Community Perspectives. J. Pharm. Heal. Serv. Res. 2020, 11 (1), 25–32. 10.1111/jphs.12327. PMC7546426 33042231

[ref67] Tindana P.; Molyneux C. S.; Bull S.; Parker M. Ethical Issues in the Export, Storage and Reuse of Human Biological Samples in Biomedical Research: Perspectives of Key Stakeholders in Ghana and Kenya. BMC Med. Ethics 2014, 15 (1), 76 10.1186/1472-6939-15-76. 25326753 PMC4210627

[ref68] Van Norman G. A. Drugs, Devices, and the FDA: Part 2. JACC Basic to Transl. Sci. 2016, 1 (4), 277–287. 10.1016/j.jacbts.2016.03.009. PMC6113340 30167516

[ref69] Cheng M. Medical Device Regulations - Global Overview and Guiding Principles; WHO Library Cataloguing-in-Publication: Geneva, Switzerland, 2003; pp 1–43. https://apps.who.int/iris/handle/10665/42744 (accessed 2020-05-03).

[ref70] World Health Organization. Advice on the Use of Point-of-Care Immunodiagnostic Tests for COVID-19 - Rapid Diagnostic Tests Based on Antigen Detection. https://www.who.int/news-room/commentaries/detail/advice-on-the-use-of-point-of-care-immunodiagnostic-tests-for-covid-19 (accessed 2021-02-23).

[ref71] European Commission Enterprise and Industry DG, Directive 98/79/EC of the European Parliament and of the Council of 27 October 1998 on in Vitro Diagnostic Medical Devices. EUR-Lex*;* 1998; pp 0001–0037. https://www.gmp-compliance.org/files/guidemgr/IVD_Directive.pdf (accessed 2021-02-23).

[ref72] The European Parliament and of the Council. Regulation (EU) 2017/746 of the European Parliament and of the Council of 5 April 2017 - On in Vitro Diagnostic Medical Devices and Repealing Directive 98/79/EC and Commission Decision 2010/227/EU; EUR-Lex*;* 2017; pp 176–332. https://eur-lex.europa.eu/eli/reg/2017/746/oj (accessed 2021-02-23).

[ref73] U.S. Food and Drug Administration (FDA). Policy for Diagnostic Tests for Coronavirus Disease-2019 during the Public Health Emergency: Immediately in Effect Guidance for Clinical Laboratories, Commercial Manufacturers, and Food and Drug Administration Staff. https://www.fda.gov/regulatory-information/search-fda-guidance-documents/policy-coronavirus-disease-2019-tests-during-public-health-emergency-revised (accessed 2020-05-01).

[ref74] Centers for Disease Control and Prevention. Clinical Laboratory Improvement Amendments (CLIA). https://www.cdc.gov/clia/index.html (accessed 2021-09-20).

[ref75] U.S. Food and Drug Administration (FDA). Quality System (QS) Regulation/Medical Device Good Manufacturing Practices. https://www.fda.gov/medical-devices/postmarket-requirements-devices/quality-system-qs-regulationmedical-device-good-manufacturing-practices (accessed 2021-05-19).

[ref76] World Health Organization, Department of Blood Safety and Clinical Technology. Current Performance of COVID-19 Test Methods and Devices and Proposed Performance Criteria*;* 2003; pp 1–43. https://ec.europa.eu/docsroom/documents/40805 (accessed 2021-05-19).

[ref77] World Health Organization, Prequalification Team - Diagnostics. Instructions for Submission Requirements: In Vitro Diagnostics (IVDs) Detecting Antibodies to SARS-CoV-2 Virus. World Health Organization, 2020; pp 1–21. https://www.who.int/diagnostics_laboratory/200703_pqt_ivd_352_v2_eul_immunoassay_requirements_ncov.pdf. (accessed 2020-12-19).

[ref78] Zhu N.; Zhang D.; Wang W.; Li X.; Yang B.; Song J.; Zhao X.; Huang B.; Shi W.; Lu R.; Niu P.; Zhan F.; Ma X.; Wang D.; Xu W.; Wu G.; Gao G. F.; Tan W. A Novel Coronavirus from Patients with Pneumonia in China, 2019. N. Engl. J. Med. 2020, 382 (8), 727–733. 10.1056/NEJMoa2001017. 31978945 PMC7092803

[ref79] Li Q.; Wu J.; Nie J.; Zhang L.; Hao H.; Liu S.; Zhao C.; Zhang Q.; Liu H.; Nie L.; Qin H.; Wang M.; Lu Q.; Li X.; Sun Q.; Liu J.; Zhang L.; Li X.; Huang W.; Wang Y. The Impact of Mutations in SARS-CoV-2 Spike on Viral Infectivity and Antigenicity. Cell 2020, 182 (5), 1284–1294. 10.1016/j.cell.2020.07.012. 32730807 PMC7366990

[ref80] Cao Y.; Su B.; Guo X.; Sun W.; Deng Y.; Bao L.; Zhu Q.; Zhang X.; Zheng Y.; Geng C.; Chai X.; He R.; Li X.; Lv Q.; Zhu H.; Deng W.; Xu Y.; Wang Y.; Qiao L.; Tan Y.; et al. Potent Neutralizing Antibodies against SARS-CoV-2 Identified by High-Throughput Single-Cell Sequencing of Convalescent Patients’ B Cells. Cell 2020, 182 (1), 73–84. 10.1016/j.cell.2020.05.025. 32425270 PMC7231725

[ref81] Ensuring Innovation in Diagnostics for Bacterial Infection Implications for Policy. European Observatory Health Policy Series*;* Morel C., McClure L., Edwards S., Goodfellow V., Sandberg D., Thomas J., Mossialos E., Eds.; European Observatory on Health Systems and Policies, 2016. PMID: 28806042. 28806042

[ref82] Metcalfe T. A. Development of Novel IVD Assays: A Manufacturer’s Perspective. Scand. J. Clin. Lab. Invest. 2010, 70, 23–26. 10.3109/00365513.2010.493361. 20515272

[ref83] Borsci S.; Kuljis J.; Barnett J.; Pecchia L. Beyond the User Preferences: Aligning the Prototype Design to the Users’ Expectations. Hum. Factors Ergon. Manuf. Serv. Ind. 2016, 26 (1), 16–39. 10.1002/hfm.20611.

[ref84] Phillips K. A.; Van Bebber S.; Issa A. M. Diagnostics and Biomarker Development: Priming the Pipeline. Nat. Rev. Drug Discovery 2006, 5 (6), 463–469. 10.1038/nrd2033. 16718275

[ref85] Berner E. S.; Graber M. L. Overconfidence as a Cause of Diagnostic Error in Medicine. Am. J. Med. 2008, 121 (5), S2–S23. 10.1016/j.amjmed.2008.01.001. 18440350

[ref86] Miller I.; Pothier K.; Dunn M. Advocacy in Personalized Medicine: A Developing Strength in a Complex Space. Pers. Med. 2010, 7 (2), 179–186. 10.2217/pme.10.2. 29783322

[ref87] Vandenberg O.; Martiny D.; Rochas O.; van Belkum A.; Kozlakidis Z. Considerations for Diagnostic COVID-19 Tests. Nat. Rev. Microbiol. 2021, 19 (3), 171–183. 10.1038/s41579-020-00461-z. 33057203 PMC7556561

[ref88] ISO/TR 10993-22:2017 Biological Evaluation of Medical Devices - Part 22: Guidance on Nanomaterials; ISO, 1st ed.; 2017. https://www.iso.org/standard/65918.html (acessed 2020-06-10).

[ref89] Budd J.; Miller B. S.; Manning E. M.; Lampos V.; Zhuang M.; Edelstein M.; Rees G.; Emery V. C.; Stevens M. M.; Keegan N.; Short M. J.; Pillay D.; Manley E.; Cox I. J.; Heymann D.; Johnson A. M.; McKendry R. A. Digital Technologies in the Public-Health Response to COVID-19. Nat. Med. 2020, 26 (8), 1183–1192. 10.1038/s41591-020-1011-4. 32770165

[ref90] Parker M. J.; Fraser C.; Abeler-Dörner L.; Bonsall D. Ethics of Instantaneous Contact Tracing Using Mobile Phone Apps in the Control of the COVID-19 Pandemic. J. Med. Ethics 2020, 46 (7), 427–431. 10.1136/medethics-2020-106314. 32366705 PMC7231546

[ref91] Morley J.; Cowls J.; Taddeo M.; Floridi L. Ethical Guidelines for COVID-19 Tracing Apps. Nature 2020, 582 (7810), 29–31. 10.1038/d41586-020-01578-0. 32467596

[ref92] Centers for Disease Control and Prevention. Interim Guidance for Antigen Testing for SARS-CoV-2. https://www.cdc.gov/coronavirus/2019-ncov/lab/resources/antigen-tests-guidelines.html (accessed 2021-02-23).

[ref93] Jensen J. M.; Raver J. L. When Self-Management and Surveillance Collide. Gr. Organ. Manag. 2012, 37 (3), 308–346. 10.1177/1059601112445804.

[ref94] Calvo R. A.; Deterding S.; Ryan R. M. Health Surveillance during Covid-19 Pandemic. BMJ. 2020, 369, m1373 10.1136/bmj.m1373. 32253180

